# Use of a novel low-fat hydrolyzed diet in the management of dogs with chronic enteropathy

**DOI:** 10.1093/jvimsj/aalag288

**Published:** 2026-11-10

**Authors:** Claudia Hernández-Anllo, Luis Feo, Marta Hervera, Jordi Puig

**Affiliations:** Internal Medicine Department, AniCura Ars Veterinària Veterinary Hospital, Barcelona 08034, Spain; Internal Medicine Department, AniCura Ars Veterinària Veterinary Hospital, Barcelona 08034, Spain; Expert Pet Nutrition, 8955 Oetwil an der Limmat, Switzerland; Internal Medicine Department, AniCura Ars Veterinària Veterinary Hospital, Barcelona 08034, Spain

**Keywords:** canine, food-responsive enteropathy, chronic enteropathy, hydrolyzed diets, low-fat diets

## Abstract

**Background:**

Dietary management plays a central role in the diagnosis and treatment of chronic enteropathy (CE) in dogs. Although hydrolyzed–protein diets are commonly used in patients with CE, and dietary fat restriction is recommended in selected gastrointestinal disorders, the combined effect of low fat and hydrolyzed protein has not been specifically evaluated.

**Hypothesis/Objectives:**

Assess clinical response and acceptability of a low-fat, hydrolyzed diet in dogs with CE.

**Animals:**

Thirty-one client-owned dogs.

**Methods:**

Prospective clinical study. Dogs were fed a commercial low-fat (2.01 g/100 kcal metabolizable energy) hydrolyzed salmon protein diet and stratified according to previous dietary history. Dogs were evaluated at baseline, 1 month, and 3 months. The primary outcome was clinical response, classified as complete response (CR), partial response (PR), or non-response (NR) based on gastrointestinal signs and the Canine Inflammatory Bowel Disease Activity Index (CIBDAI) score. Dogs with CR or PR were considered responders. Diet acceptability was assessed.

**Results:**

At T1, 20 of 31 (64.5%) dogs were categorized as CR, 3 of 31 (9.7%) as PR, and 8 of 31 (25.8%) as NR. Twenty-three of 31 (74.2%) dogs had a positive clinical response. The CIBDAI scores decreased significantly from T0 to T1 (*P* < .001). At T2, 94.4% of initial responders maintained CR. Diet acceptability was excellent in 75.0% of cases.

**Conclusions and clinical importance:**

This low-fat, hydrolyzed diet had significant short- and mid-term effectiveness and was well tolerated in dogs with CE, reinforcing its potential as a treatment for food-responsive enteropathy.

## Introduction

Chronic enteropathy (CE) in dogs encompasses persistent or recurring gastrointestinal signs lasting more than 3 weeks,^1–3^ driven by host susceptibility, barrier dysfunction, immune dysregulation, diet, and alterations in the microbiota.^2^^,^^4^ Chronic enteropathy is commonly diagnosed and subclassified based on treatment response into food-responsive (FRE), antibiotic-responsive (ARE), immunosuppressant-responsive (IRE), and non-responsive enteropathy (NRE).^5^ Emerging evidence has challenged the clinical value of the traditional ARE category and led to a clear shift away from routine antibiotic trials in dogs with CE^1^ and introduced the concept of microbiota–related modulation–responsive enteropathies.^2^

A favorable clinical response to a dietary trial supports the diagnosis of FRE. Usually, multiple diets using different strategies, such as highly digestible, hydrolyzed, single-source novel protein, or fiber-rich diets, are used before a patient is classified as non-responsive to dietary intervention.^2–4^ Although differences exist across studies, dogs with FRE tend to be younger and show milder clinical signs than those with other types of CE.^4^^,^^6^^,^^7^ Decreasing fat levels is described to be potentially beneficial in various gastrointestinal conditions, including acute gastroenteritis,^8^ gastrointestinal motility disorders,^9^ pancreatitis,^10^ and conditions associated with intestinal lymphangiectasia.^11^ Low-fat or ultra-low-fat diets benefit some dogs with protein-losing enteropathies (PLE) by decreasing lymphatic flow and lacteal dilatation, thereby decreasing protein leakage.^12^

Responses to dietary interventions in dogs with CE vary considerably among different studies. Response rates of 52.6%^13^ and 55.7%^6^ have been reported with a non-hydrolyzed diet based on a single protein source containing approximately 4.5 g/100 kcal metabolizable energy (ME) of fat. Similarly, favorable responses have been reported, including a hydrolyzed—protein diet also providing 4.5 g/100 kcal ME of fat, in which 79% of dogs achieved complete clinical remission and an additional 21% achieved a partial response (PR) at the end of the study.^14^ Another study using the same diet reported clinical remission in 69% of dogs.^15^ In a pilot study, a diet with 4.3 g/100 kcal ME of fat achieved clinical remission in 61.5% of dogs after 5 weeks, increasing to 91% after 10 weeks.^16^ In contrast, other studies have evaluated diets with comparatively lower fat content, including an elemental diet containing 2.79 g/100 kcal ME of fat, which achieved a response rate of 68%.^17^ This variability highlights nutrition’s key role in managing CE in dogs and shows the disease’s heterogeneity. Differences in study design, populations, severity, protein source, fat content, and the use of hydrolyzed or novel protein formulations account for the variation in response rates.

Although both hydrolyzed-protein and low-fat diets have been studied independently and implemented in clinical practice, the combination of these 2 dietary strategies has not been assessed previously in dogs with CE. Our objectives were to evaluate the clinical response and diet acceptability of a low-fat, hydrolyzed diet in dogs with CE. We hypothesized that feeding a low-fat, hydrolyzed diet would result in significant clinical improvement in dogs with CE, with a response rate exceeding 50%.

## Materials and methods

A prospective cohort study was conducted at AniCura Ars Veterinària Veterinary Hospital, involving client-owned dogs diagnosed with CE between September 2022 and November 2024. Chronic enteropathy was defined as persistent or recurrent gastrointestinal signs lasting longer than 3 weeks, and a case-specific assessment was performed to rule out extra-intestinal and other intestinal conditions, including structural disease, infectious, or metabolic causes.^3^ The minimum diagnostic evaluation is detailed in the Appendix.

Dogs that had not previously been fed the study diet were eligible for inclusion. Dogs diagnosed with PLE were eligible. Gastrointestinal histopathology was documented when available. Prior diet trials, details of supplements and medical treatments administered before or during the study are provided in the Appendix.

Dogs were stratified into 2 groups based on whether they had previously undergone hydrolyzed or novel-protein trials (Group A) and whether they received dietary trials featuring at least one hydrolyzed commercial or single-source novel protein diet (Group B; Appendix). The chosen trial test diet was the hydrolyzed salmon, low-fat formulation of SPECIFIC Digestive Support Low Fat CID—LF (Dechra Veterinary Products SL; Table 1) fed exclusively during the study assessment period.

**Table 1:** Nutritional content of the diet and feeding instructions.

	g/ 100 g (as fed)	g/ 100 g (dry matter)	g/ 100 kcal ME
	1371 kJ/ 100 g (328 kcal/ 100 g)	1507 kJ/ 100 g (360 kcal/ 100 g)	-
**Moisture**	9.0	–	–
**Protein**	23	25.3	7.02
**Fat**	6.6	7.3	2.01
**Carbohydrate**	49.7	54.6	15.2
**Crude fiber**	3.7	4.1	1.14
**Calcium**	0.99	1.1	0.3
**Phosphorus**	0.51	0.56	0.16
**Sodium**	0.35	0.38	0.11
**Omega 3**	1.51	1.66	0.46
**Relation n-3:n-6**	1:1	1:1	1:1
**List of ingredients**	Tapioca, rice protein, hydrolyzed salmon protein, minerals, fish oil, potato protein, hydrolyzed animal protein, vegetable oil, beet pulp, vitamins and trace elements, cellulose powder, yeast, psyllium husk, fructo-oligosaccharides, AuraGuard (calcium carbonate, citrus extract, maltodextrin, sodium chloride).		
**Feeding instructions**	Caregivers were advised to gradually introduce the new diet to their pets over 5 to 7 days and feed it exclusively during the study. Feeding quantities were based on the manufacturer’s instructions and adjusted to ideal body weight.		

The boldface was used to highlight the relevant nutritional components and other diet-related information provided by the manufacturer (macronutrients, selected minerals and fatty acid parameters, the ingredient list and feeding instructions).

Patient data were collected at baseline (T0), 1 month (T1), and 3 months (T2). Clinical evaluation data evaluated at each time point are detailed in the Appendix. Among clinical variables, the Canine Inflammatory Bowel Disease Activity Index (CIBDAI) score was calculated.^18^ Further categorization was made based on the recurrence pattern of clinical signs (Table 2).

**Table 2:** Definitions of recurrence pattern of clinical signs.

**Unchanged**	The patient exhibits the same frequency of clinical signs as baseline reported frequency.
**Worsened**	The patient experiences a higher frequency of clinical signs than as baseline reported frequency.
**Improved**	The patient exhibits one episode or less than one in a month.
**Resolved**	The patient no longer exhibits clinical signs.

The primary outcome was the clinical response to the diet at T1, measured by the CIBDAI score and the caregiver’s impression of adequate relief of gastrointestinal clinical signs. Patients were categorized into 3 response categories based on their clinical response (Table 3).^19^ Dogs that were lost to follow-up or had their diet discontinued for non-clinical reasons were analyzed up to the last available time point. Patients with CR and PR at T1 continued the diet trial through T2. Dogs classified as non-response (NR) at T1 were excluded from subsequent analysis at T2. Data collected at T2 were summarized descriptively. For patients categorized as NR at T1 or T2, a definite diagnosis was recorded based on outcome. The recurrence pattern of clinical signs was recorded. The secondary outcome measure was diet acceptance, detailed in the Appendix. Data collected at each time point are presented in Table 4. Statistical methods are detailed in the Appendix.

**Table 3:** Definitions of clinical category response.^19^

**Complete response (CR)**	Resolution of clinical signs or significant improvement for the caregiver associated with a CIBDAI reduction of > 75% from baseline.
**Partial response (PR)**	Improvement but not complete resolution of clinical signs or stability for the caregiver associated with a reduction of 25—75% of baseline CIBDAI.
**Non-response (NR)**	No significant changes in clinical signs for the caregiver associated with a reduction of < 25% of CIBDAI score from baseline.
The positive clinical response group included all patients who achieved CR or PR.	

The boldface is used to identify the clinical response categories (indicate the names used in the paper), which are then defined in the adjacent column. Abbreviation: CIBDAI = Canine Inflammatory Bowel Disease Activity Index.

**Table 4:** Data collection at each time point.

Variable	T0 (Baseline)	T1 (1 month)	T2 (3 months)
**Signalment**	*		
**Gastrointestinal clinical signs**	*	*	*
**Body Weight (BW)**	*	*	*
**Body Condition Score (BCS)**	*	*	*
**CIBDAI score**	*	*	*
**Recurrence pattern of clinical signs**		*	*
**Diet acceptability**		*	*
**Laboratory and imaging work-up**	*		
**Gastrointestinal histopathology**	*		

^*^Data collected at each time-point. The boldface is used to identify the clinical variables included in the data collection at each time point.

## Results

### Study population and T0 assessment

Initially, 32 patients were evaluated and started the diet. One dog refused to eat and was excluded from the clinical efficacy assessment but included in the diet acceptability analysis. Therefore, 31 dogs met the inclusion criteria and were enrolled in the clinical efficacy analysis. Eleven of 31 (35.5%) patients were included in group A, and 20 of 31 (64.5%) in group B.

In the overall population, the median age was 6 years (range: 1-13), 4 years (range: 1-13) in group A, and 6.5 years (range: 1-11) in group B. Seventeen of 31 (54.8%) dogs were female (2 intact, 15 spayed), and 14 of 31 (45.2%) were male (5 intact, 9 neutered). In group A, 6 of 11 (54.5%) dogs were females, all of them spayed, and 5 of 11 (45.5%) dogs were males (1 intact, 4 neutered). In group B, 12 of 21 (57.1%) were females (3 intact, 9 spayed), and 9 of 21 (42.9%) were males (4 intact, 5 neutered). The median body weight (BW) was 19 kg (range: 2.0-48.0 kg), 19 kg for group A (range: 3.8-37.9 kg) and 16.35 kg for group B (range: 2.0-48.0 kg). Median body condition score (BCS) at T0 was 5 out of 9 (range: 2-7 of 9), 5 for group A and B (range: 4-6 of 9 and 2-7 out of 9, respectively). The most represented breeds were mixed (6 of 31), followed by Golden Retriever (4 of 31), French Bulldog (2 of 31), Spanish Water Dog (2 of 31), Yorkshire Terrier (2 of 31), and West Highland White Terrier (2 of 31). Thirteen other breeds were represented by a single individual each. The median duration of clinical signs before inclusion was 24 weeks (range: 3-240 weeks), 32 weeks (range: 4-192) in group A, and 24 weeks (range: 3-240) in group B. Baseline clinical signs details are summarized in Table 5. At baseline (T0), no significant differences were observed between groups A and B for age (*P* = .89), sex (*P* = 1.00), BW (*P* = .75), BCS (*P* = .23), time since onset (*P* = .47), duration of clinical signs (*P* = .47), and fecal type (*P* = 1.00).

**Table 5:** Baseline clinical signs details.

Variable	Overall population (*n*; %)
**Diarrhea**	24 (74.4 %)
**Vomiting**	21 (67.4 %)
**Hyporexia**	12 (38.7 %)
**Nausea**	5 (16.1 %)
**Pruritus**	4 (12.9%)
**Hematochezia**	3 (9.7 %)
**Regurgitation**	3 (9.7 %)
**Weight loss**	2 (6.5 %)
**Pica behavior**	1 (3.2 %)
**Small intestinal diarrhea**	7 (22.6 %)
**Large intestinal diarrhea**	10 (32.3 %)
**Mixed diarrhea**	6 (19.4 %)
**Normal feces**	8 (25.8 %)

Among the overall population, the median number of previously prescribed diets was 1 (range: 0-5). In group A, 6 of 11 (54.5%) dogs had received a highly digestible veterinary diet; none had received a low-fat formulation. In group B, 10 of 20 (50.0%) dogs had been fed a highly digestible diet, and 2 of 20 (10.0%) dogs had also received a low fat formulation. In this group, 12, 6, and 2 of 20 dogs were exposed to 1, 2, and 3 diets of a hydrolyzed or single-source novel protein, respectively. A summary of previous diet trials is presented in Table 6, and the number of dietary trials for each nutritional strategy for each group is summarized in Table 7.

**Table 6:** Previous dietary trials for each group.

	Group A (*n* = 11)	Group B (*n* = 20)
**Highly digestible diet**	6	10
**Low-fat diet**	0	2
**Hydrolyzed/novel protein diet**	N/A	20

Abbreviation: N/A = not applicable.

**Table 7:** Number of diets previously tried by each nutritional strategy within each group.

Number of dietary trials	Group A (*n* = 11)	Group B (*n* = 20)	Group A (*n* = 11)	Group B (*n* = 20)	Group A (*n* = 11)	Group B (*n* = 20)
	Highly digestible		Low-fat		Hydrolyzed/single-source novel protein	
**0**	5	10	11	18	N/A	0
**1**	4	7	0	2	N/A	12
**2**	2	3	0	0	N/A	6
**3**	0	0	0	0	N/A	2

Abbreviation: N/A = not applicable.

In the blood testing evaluation at T0, 2 dogs showed abnormalities in the CBC; 1 had mild non-regenerative anemia, and another dog had mild neutropenia, both of which were included in group B. No enrolled dogs met the PLE criteria at baseline. One dog had a moderate increase of alkaline phosphatase activity and mild hypertriglyceridemia, whereas another dog had mild hypercholesterolemia. Serum cobalamin concentrations were measured in 25 of 31 (80.6%) dogs at T0. Based on the laboratory reference range of 275-590 ng/dL, 4 of 25 (16.0%) had decreased cobalamin concentrations, including 1 patient with undetectable concentrations (<150 ng/dL). Eight dogs (32.0 %) had results within the reference range, whereas 13 of 25 (52.0%) dogs had concentrations exceeding the upper limit of the reference range. Among the hypocobalaminemic patients, 3 were classified in group B and one in group A. All hypocobalaminemic dogs were supplemented with cyanocobalamin (Optovite B_12_ 1 mg/2 mL, Normon) either parenterally once weekly for 6 consecutive weeks, or orally with 0.025 mg/kg for dogs < 20 kg BW, and 1 mg of cyanocobalamin for dogs > 20 kg BW.^20^

The median CIBDAI score in the overall population before starting the diet trial was 5 (range: 2-11). In group A, the median score was 4 (range: 3-7), whereas in group B it was 5.5 (range: 2-11). Six of 31 (19.4%) patients had CIBDAI scores of 0-3 (not clinically relevant), 4 in group A and 2 in group B. Twelve patients (12 of 31; 38.7%) had scores of 4-5 (mild disease), including 4 of 12 in group A and 8 of 12 in group B. Eleven of 31 (35.5%) patients had scores of 6-8 (moderate disease), with 3 of 11 in group A and 8 of 11 in group B. Finally, 2 of 31 (6.5%) patients had CIBDAI scores of 9 or higher (severe disease), both were in group B. No significant differences were observed in median CIBDAI scores at T0 between groups A and B (*P* = .11).

Endoscopic gastrointestinal biopsy samples were obtained from 6 of 31 patients: 1 in group A and 5 in group B. Gastroduodenoscopy was performed in 4 patients, and in 2 patients, both gastroduodenoscopy and ileocolonoscopy were performed. Histopathologic evaluation identified mild-to-moderate lymphoplasmacytic inflammation in at least one gastrointestinal segment in all cases. Additionally, 2 dogs exhibited eosinophilic inflammation (2 in the duodenum and 1 in the ileum), and 1 dog showed histopathologic evidence of superficial lacteal dilatation.

### Clinical response at T1

At T1, 31 patients were evaluated. Of these, 24 of 31 (77.4%) dogs had CIBDAI scores of 0-3 (not clinically relevant), including 9 from group A and 15 from group B. Four of 31 (12.9%) patients had scores of 4-5 (mild), all of which were in group B. Three of 31 (9.7%) patients had scores of 6-8 (moderate), with 2 in group A and 1 in group B. No dogs had CIBDAI scores of 9 or higher (severe) at T1. When comparing CIBDAI scores between the 2 time points of the overall population, a significant decrease was observed from T0 to T1 (*P* < .001; Figure 1). The same tendency was observed for CIBDAI scores in group A (*P* = .01) (Figure 2) and in group B (*P* < .001; Figure 3). Based on response classification groups, 64.5% of patients (20 of 31) achieved CR, 9.7% (3 of 31) PR, and 25.8% (8 of 31) NR. Overall, a positive clinical response was observed in 23 of 31 (74.2%), with 7 of 11 (63.6%) in group A and 16 of 20 (80.0%) in group B. A schematic representation of the response proportion at each time point from each group is presented in Figure 4. The 8 patients in the NR group were categorized according to the definitive diagnosis: 3 were classified as FRE because of a clinical response to a different dietary trial with an alternative food. Of this subset of dogs, 1 responded to a hydrolyzed protein diet, and 2 responded to a single-source novel protein diet. Of the remaining patients, 1 was classified as IRE, 1 as NRE, and 3 were lost to follow-up. Considering the overall response in our study and the dietary response beyond the study's time points among dogs with available follow-up, 26 of 31 (83.9%) patients responded to the diet.

**Figure 1:**
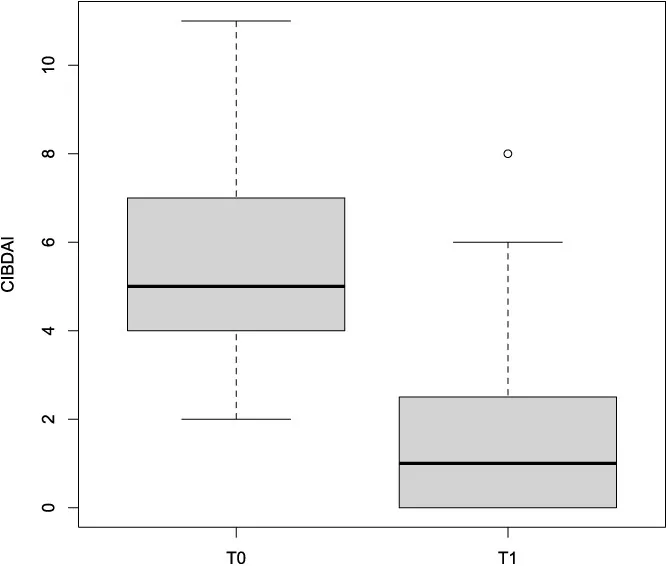
CIBDAI distribution in the overall population between T0 and T1. Median CIBDAI scoring of the overall population at T0 is 5 (range: 2-11) and at T1 is 1 (range: 0-8). There were significant differences between median CIBDAI scoring between T0 and T1 (*P* < .001).

**Figure 2:**
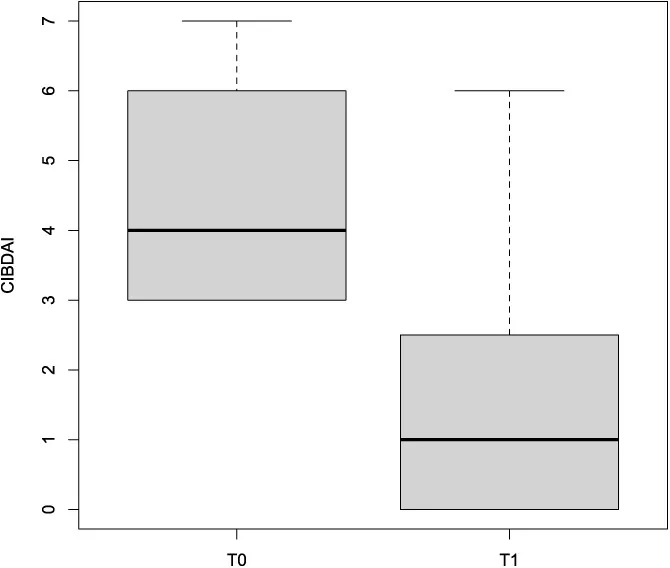
CIBDAI distribution group a between T0 and T1. Median CIBDAI scoring of group A at T0 is 4 (range: 3-7) and at T1 is 1 (range: 0-6). There were significant differences between median CIBDAI scoring between T0 and T1 (*P* = .006).

**Figure 3:**
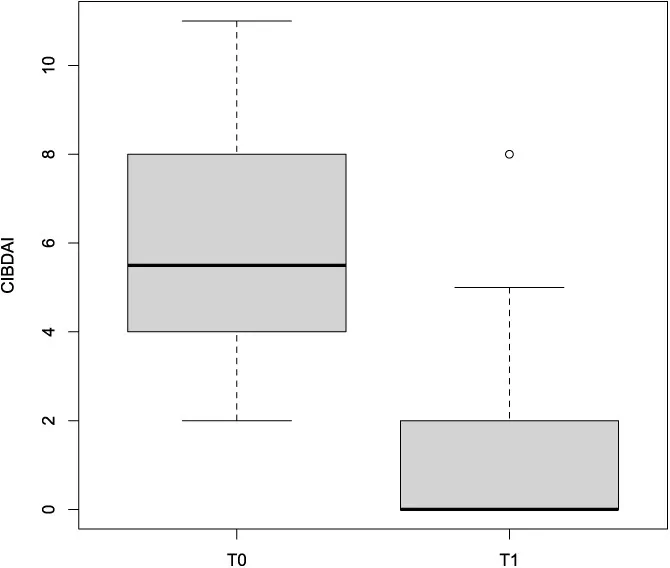
CIBDAI distribution group B between T0 and T1. Median CIBDAI scoring of group B at T0 is 5.5 (range: 2-11) and at T1 is 0 (range: 0-8). There were significant differences between median CIBDAI scoring between T0 and T1 (*P* < .001).

**Figure 4:**
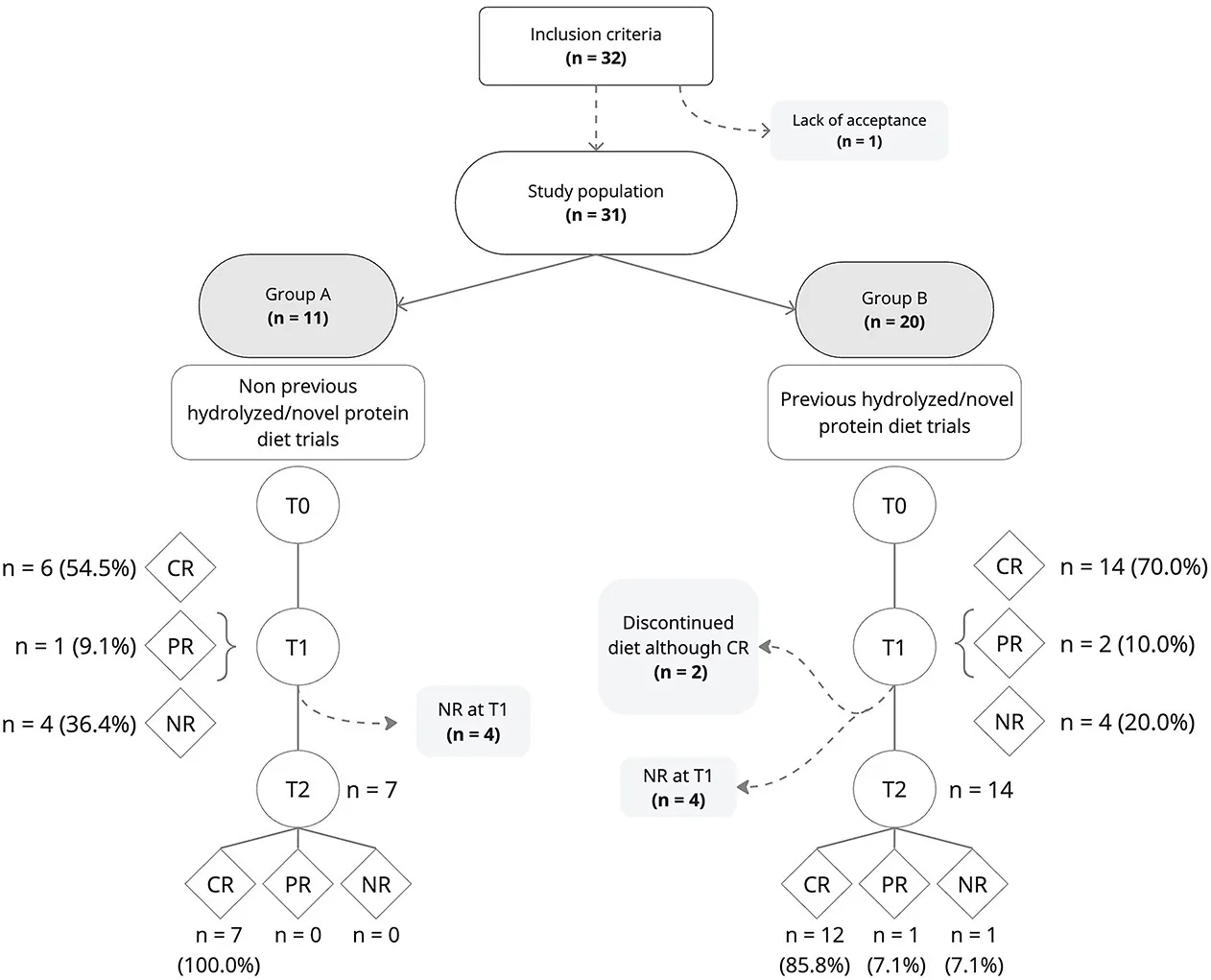
Schematic overview of the study and response groups.

Table 8 summarizes the recurrence pattern of clinical signs. In the overall population, the recurrence pattern of clinical signs resolved in 15 of 31 (48.4%) patients, remained unchanged in 9 of 31 (29.0%) dogs, improved in 4 of 31 (12.9%) and worsened in 3 of 31 (9.7%). No significant differences in the recurrence patterns were found among the groups (*P* = .59).

**Table 8:** Recurrence pattern of clinical signs evolution between baseline (T0) and T1.

Recurrence pattern	Overall (*n* = 31)	Group A (*n* = 11)	Group B (*n* = 20)
**Resolved**	15 (48.4 %)	4 (36.4 %)	11 (55.0 %)
**Unchanged**	9 (29.0 %)	4 (36.4 %)	5 (25.0 %)
**Improved**	4 (12.9 %)	2 (18.2 %)	2 (10.0 %)
**Worsened**	3 (9.7 %)	1 (9.1 %)	2 (10.0 %)

No significant associations were identified between response classification groups at T0 and clinical variables including age (*P* = .08), sex (*P* = .40), BW (*P* = .78), BCS (*P* = .16), duration of clinical signs (*P* = .70), fecal type (*P* = .58), group allocation (A or B; *P* = .72), number of formulations previously trialed (*P* = .43), and baseline CIBDAI score (*P* = .87).

### Outcome at T2

At T2, 21 of 31 (67.7%) dogs were available for assessment. Among the 18 dogs categorized as CR at T1 and maintained on the study diet through T2, 17 (94.4%) remained as CR at T2, indicating sustained clinical improvement in most initial CR patients. The 10 patients not evaluated at this time point were excluded: 8 because of lack of response at T1, and 2 because caregivers preferred an alternative diet despite clinical improvement (both CR at T1, with CIBDAI scores of 0 at T1).

Among the 3 patients classified as PR at T1, 2 achieved CR at T2 (1 from group A and 1 from group B), whereas 1 patient from group A remained PR at T2. One patient in group B transitioned from CR at T1 to NR at T2. This dog had a baseline CIBDAI score of 11, improved to 1 at T1, and subsequently relapsed to 11 at T2. This patient, classified as NR at that time point, subsequently was classified as FRE after responding to another dietary trial with a soy-based hydrolyzed protein diet. Figure 4 presents a schematic representation of the response proportion at each time point.

The CIBDAI scores remained stable or improved in most dogs between T1 and T2. Thirteen of 21 dogs (61.9%) experienced no change in CIBDAI scoring, 4 (19.0%) dogs showed a decrease in CIBDAI score, and 4 (19.0%) dogs showed an increase. Based on CIBDAI categories, 20 of 21 (95.2%) dogs showed “insignificant disease” at both time points, whereas 1 of 21 (4.8%) dogs showed worsening of CIBDAI category from “insignificant disease” to “severe disease.”

Clinical signs recurrence pattern improved between the 2 time points in 15 of 21 (71.4%) dogs, whereas 4 of 21 (19.0 %) experienced worsening. Ten of 21 (47.6%) patients evaluated at T2 had resolved frequency of clinical signs at T1, which was sustained at T2. Three of 21 (14.3%) patients had unchanged clinical sign frequency at T1, which resolved at T2. One of 21 (4.8%) had an improved frequency of clinical signs at T1 that resolved at T2, and another dog (4.8%) remained in the improved category at both time-points. Among the 4 dogs that experienced a worsening recurrence pattern, all had an initial improvement from baseline, with a resolved recurrence pattern of clinical signs at T1, but 3 (14.3%) experienced worsening with an improved recurrence pattern at T2, whereas the remaining patient (4.8%) changed from an improved to an unchanged recurrence pattern of clinical signs at T2. Table 9 presents detailed transitions between recurrence categories for each patient.

**Table 9:** Association between response and recurrence pattern.

Patient number	One–month follow-up (T1)		Three-month follow-up (T2)	
	Response group	Recurrence pattern	Response group	Recurrence pattern
**Group A**				
**1**	PR	Unchanged	CR	Resolved
**2**	CR	Resolved	CR	Improved
**3**	CR	Resolved	CR	Resolved
**4**	CR	Resolved	CR	Resolved
**5**	NR	Unchanged	–	–
**6**	CR	Resolved	CR	Resolved
**7**	CR	Improved	CR	Improved
**8**	CR	Improved	CR	Resolved
**9**	NR	Unchanged	–	–
**10**	NR	Worsened	–	–
**11**	NR	Unchanged	–	–
**Group B**				
**1**	CR	Unchanged	CR	Resolved
**2**	CR	Resolved	CR	Resolved
**3**	CR	Resolved	CR	Resolved
**4**	NR	Worsened	–	–
**5**	PR	Resolved	PR	Improved
**6**	CR	Unchanged	CR	Improved
**7**	CR	Resolved	CR	Resolved
**8**	NR	Worsened	–	–
**9**	CR	Resolved	CR	Improved
**10**	CR	Resolved	CR	Resolved
**11**	CR	Resolved	CR	Resolved
**12**	CR	Resolved	CR	Resolved
**13**	CR	Improved	CR	Resolved
**14**	CR	Improved	NR	Unchanged
**15**	PR	Unchanged	CR	Resolved
**16**	CR	Resolved	CR	Resolved
**17**	NR	Unchanged	–	–
**18**	NR	Unchanged	–	–
**19**	CR	Resolved	–	–
**20**	CR	Resolved	–	–

Baseline cobalamin concentrations were not associated with response category: 2 of 4 patients with hypocobalaminemia achieved CR, whereas the other 2 were in the NR category at both follow-up time points.

### Diet acceptability

Diet acceptability was rated as excellent in 24 of 32 (75.0%) dogs, moderate in 6 of 32 (18.8%) cases, and poor in 1 of 32 (3.1%). One patient initially assigned to group B was withdrawn from the clinical efficacy assessment because of complete reluctance to eat the diet (1 of 32; 3.1%). In group A, 9 of 11 (81.8%) dogs had excellent acceptance, whereas 2 of 11 (18.2 %) dogs had moderate acceptance. In group B, excellent acceptance was observed in 15 of 20 dogs (75.0%), moderate in 4 (20.0%), and poor in 1 (5.0%). The patient with poor acceptance was withdrawn from the diet because of the caregiver's decision, despite having CR at T1.

## Discussion

In our prospective uncontrolled clinical study, feeding a low-fat, hydrolyzed diet was associated with clinical improvement in dogs with CE. A positive clinical response was observed in 74.5% of dogs after 1 month, and 94.4% of initial responders maintained this improvement at 3 months. Among patients that responded to food with an alternative diet beyond the study evaluation, the response rate was 26 of 31, resulting in an overall food response of 83.9% of our population, reinforcing the concept that diet is paramount in managing most forms of CE. Diet acceptability assessment also indicates good to excellent overall acceptance of the diet. These findings indicate that a low-fat, hydrolyzed diet may contribute to favorable clinical outcomes in dogs with CE, emphasizing the importance of dietary strategies in the management of this condition.

Several studies have reported similar outcomes, and our findings are consistent with the latest research on the clinical efficacy of dietary trials in dogs with CE. A positive response (83.0%) was observed in dogs diagnosed with non-PLE CE when maintained on a fish-based hydrolyzed diet for 12 weeks, as assessed using the Canine Chronic Enteropathy Activity Index (CECCAI).^21^ In that study, the authors found the same clinical response with the hydrolyzed fish diet as with the single-source novel protein diet used in the control group, and thus it is unclear whether the benefit was a result of the hydrolyzed nature of the diet or related to the general advantage of a dietary trial. Another study using an elemental diet, evaluated using the CECCAI, found that 73.0% of dogs achieved clinical remission.^17^ As in our study, the clinical response was rapid and sustained, but the follow-up was shorter at 8 weeks than at 3 months in our study. The elemental diet used had a moderate fat content, higher than the 2.01 g/100 kcal ME in our study, and a lower fat intake in some patients compared with their previous diet. Another study reported a response rate of 69.0% based on CECCAI scores, after using a soy-based hydrolyzed diet for 2 weeks.^15^ Lastly, in 3 prospective studies using the same protein source (salmon) as in our study, but as a single-source protein rather than a hydrolyzed formula, the response rate was 55.7%,^6^ and 52.6%.^13^ The variable response rates reported in these studies are affected by both the inherent complexity of CE and differences in study design. These differences encompass the type of diet, including various protein sources, hydrolyzed versus non-hydrolyzed formulations, and variations in fat content and potential digestibility. Such factors pose substantial obstacles to making direct comparisons among studies.

Hydrolyzed diets prevent immune detection in sensitized patients by breaking down protein structures and removing immunogenic epitopes.^22^ Despite the potential benefits of hydrolyzed diets over single-source novel protein diets, including the need for a less thorough diet history or decreased antigenicity, studies directly comparing these 2 diet strategies are limited.^22^^,^^23^ In addition, the benefits of dietary management extend beyond decreasing antigenic stimulation, because other factors that benefit the patient include increased digestibility, the immunomodulatory effects of omega-3 fatty acids, decreased fat content^24^ and effects on the gastrointestinal microbiota, among others.^15^ Affected patients show changes compatible with dysbiosis, as evidenced by lower microbial diversity^25^^,^^26^ and significantly higher dysbiosis index values.^25^ Specifically, dogs that had experienced a clinical response to the diet also had increased amounts of *Peptacetobacter hiranonis*, increased fecal concentrations of secondary bile acids, and ameliorated gut dysbiosis compared with their pre-trial status.^15^

In our study, all but 2 dogs experienced a decrease in fat intake compared with previous diet trials. Multiple mechanisms could explain the positive effects of decreasing dietary fat intake in dogs with CE. In a prospective study, a highly digestible, low-fat diet after metronidazole treatment was found to support the recovery of microbial populations and metabolism compared with dogs that received the control diet, resulting in increases in fecal short-chain fatty acids and fecal IgA concentrations, and decreases in fecal sterol and fatty acid concentrations.^27^ High-fat diets have been shown to impair the mucosal barrier in dogs, altering fecal bacterial populations, transiently increasing colonic permeability,^28^ modifying the gastrointestinal microbiome and compromising epithelial integrity. These factors affect mucosal permeability, facilitating the translocation of lipopolysaccharides and contributing to low-grade systemic inflammation.^29^ Altered gastrointestinal function may impair fat digestion, resulting in increased amounts of fat reaching the colon. This excess fat may contribute to dysbiosis and stimulate fluid secretion, leading to osmotic diarrhea.^23^ Additionally, low-fat diets may promote faster gastric emptying, which could help alleviate clinical signs such as nausea and vomiting in affected dogs. The coexistence of pancreatitis and CE was documented in a retrospective study, supporting the potential advantages of a combined hydrolyzed and low-fat diet in certain patients.^10^ Possible explanations include the production of pancreatic autoantibodies in dogs with CE, as occurs in some people with Crohn’s disease.^30^ In the mentioned study, dogs with high canine pancreatic lipase immunoreactivity (cPLI) were less responsive to dietary changes and showed limited improvement with corticosteroid treatment. However, the dietary trials in this group were labeled as “hypoallergenic” or “novel protein,” and none described a low-fat formulation. In our study, cPLI was not routinely measured, and thus we were unable to examine its possible association with a low-fat diet intervention. Decreasing fat intake may offer an additional advantage by decreasing the delivery and colonic accumulation of primary bile acids. This effect could be particularly relevant in the context of decreased activity of bile acid–converting bacteria such as *P. hiranonis*. In this setting, a microbiota shift driven by a hydrolyzed protein source, combined with a lower-fat formulation, may produce a synergistic effect. Despite our study’s suggested benefits, it is not possible to determine whether the clinical response results from an isolated effect of each dietary strategy or from a combined effect, because no control group was included. Interestingly, 2 dogs that previously had consumed a low-fat diet and at least one hydrolyzed formulation each achieved CR during the study period.

Approximately one-quarter of the patients in our study were assigned to the NR group. Notably, 4 of the 9 NR dogs subsequently were classified as FRE after being fed another diet (a hydrolyzed protein diet or a single-source novel protein diet) after being withdrawn from the study, supporting the idea that, in some cases, more than one elimination trial should be conducted before considering a patient non-food responsive.^4^^,^^5^^,^^12^^,^^16^^,^^21^^,^^31^^,^^32^ One patient was categorized as IRE and another as NRE, consistent with the expected prevalence of clinical categories in CE.

No significant associations were observed among signalment, body condition score, disease activity, or other clinicopathological findings and clinical response. Traditionally, FRE-affected dogs have been reported more commonly to be younger, have milder disease activity scores, and suffer predominantly from large-bowel diarrhea.^2^^,^^6^^,^^7^^,^^15^^,^^33^ Despite reports suggesting that patients with increased disease severity are less likely to respond to a dietary trial,^6^^,^^7^^,^^22^^,^^33^ our study was unable to confirm these findings, because baseline CIBDAI did not differ across response categories. The most likely explanation for this lack of differences might be the small sample size and population, and the fact that most patients had low baseline CIBDAI scores, with only 2 of 31 patients (6.5%) classified as having “severe disease.” Although the sole relapsing patient had the highest baseline CIBDAI score, the other dog with a baseline CIBDAI score classified as “severe disease” achieved CR at both time points, thereby corroborating previous observations that dogs with FRE also can present with severe disease manifestations.^31^ Additionally, none were categorized as a PLE. Hypoalbuminemia previously has been stated to be a negative prognostic factor and associated with refractoriness to treatment.^6^^,^^34^ Fat restriction is a mainstay of management for primary and secondary lymphangiectasia.^11^ Only one patient, that achieved CR in the first month, showed histologic evidence of superficial lacteal dilatation, even though the dog presented without baseline hypoalbuminemia.

In some cases, discordance was noted between the assigned response category and the evolution of the recurrence pattern of clinical signs. The CIBDAI scoring system has limitations in assessing the frequency of clinical signs. It accounts for changes in the frequency of vomiting and diarrhea, but no other gastrointestinal clinical signs, and requires relatively large changes in frequency to show a score change. In our study, assessing the recurrence pattern of clinical signs helped us to understand individual changes in frequency and provided better insight into the progression of each case. Based on our results, although some patients with improved CIBDAI scores continued to experience recurrent clinical signs, dietary treatment led to less severe flare-ups, likely improving the patients’ quality of life. A direct link has been observed between the severity of CE and decreased quality of life in affected dogs, and any improvements in disease severity are likely to be associated with a meaningful positive effect on affected dogs.^32^^,^^35^

The second objective of our study was to assess the acceptance of a new low-fat diet in dogs diagnosed with CE. Diet acceptance was rated excellent in most cases (75.0%), with only 1 dog completely refusing to eat the diet, even among dogs that did not respond clinically or had higher CIBDAI scores. This is a notable finding, given that hydrolyzed diets often are regarded as less palatable because of flavor changes from peptides and amino acids,^36^ and decreased fat content can further decrease acceptability. Despite these potential limitations, the high diet acceptability observed in our study supports the practical use of this formulation in a clinical setting, even in inappetent patients.

Limitations of our study include its single-center, non-randomized, uncontrolled trial design. It lacked comparison groups of dogs on a standard hydrolyzed non-low-fat diet or a low-fat diet, preventing isolation of the effects of these separate dietary strategies. Despite the study’s prospective clinical design, the diagnostic investigation of individual dogs occasionally was influenced by clinical decisions and caregivers’ financial considerations. Although all dogs underwent a consistent minimum diagnostic evaluation to support a CE diagnosis, diagnostic protocols were not identical in every case. Another limitation is that we could not systematically determine the fat content of all previous diets from the available dietary histories, particularly for home-prepared diets and single-source novel-protein formulations. This limitation reflects the challenges of obtaining complete dietary histories in clinical practice, because caregivers may not fully recall previously fed diets.^37^ Among the commercial diets we could reliably identify, most had higher reported fat concentrations than the study diet, and none were specifically labeled as low-fat, but we cannot rule out the possibility that some dogs previously received diets with fat content comparable to the study diet. The temporary assessment lasted only 3 months, and a longer follow-up would have been beneficial for evaluating long-term use of the diet. However, most studies reporting the clinical efficacy of dietary trials in dogs with CE have assessment periods of similar or shorter duration. The small sample size limits the power of subgroup analyses, especially for some clinical presentations, such as hypocobalaminemic patients. The primary outcome measure relied on both a validated clinical scale (CIBDAI) and caregiver impressions of relief from clinical signs, with the latter being highly subjective. To address the stated limitations of the CIBDAI score in assessing the full clinical picture in some cases, we compared the category response with the subjective impression of relief of clinical signs, along with descriptions of recurrence of clinical signs at each time point. Furthermore, most patients were not evaluated for gastrointestinal histopathology because dietary trials are typically the initial step in diagnosing and treating suspected CE, and gastrointestinal biopsies are generally not recommended in stable patients until multiple dietary trials have failed.^2^^,^^4^ In addition, other diseases, such as gastrointestinal neoplasia, were unlikely in the study population. Lastly, no measurements of specific dysbiosis indicators, such as the baseline quantitative polymerase chain reaction dysbiosis index^38^ at baseline and follow-up, or changes in bile acid profiles, were carried out. These would have been valuable tools to support the suspected effects on microbiome structure and bile acid metabolism in these patients. Although the uncontrolled study design prevents precise differentiation of the specific impact of fat restriction from that of hydrolyzed protein, our findings indicate that this dietary strategy can effectively manage dogs with CE. The results of our study should be viewed as hypothesis-generating and additional larger, controlled, and more comprehensive comparative studies should be carried out.

## Conclusion

Our findings support the use of a low-fat hydrolyzed diet as a valuable therapeutic option for dogs with CE, with positive short- and mid-term responses and excellent diet acceptability. The observed response rate was comparable to that reported for other hydrolyzed formulations, supporting its use as a potential first-line strategy for FRE patients. Additional randomized controlled studies are warranted to confirm long-term efficacy and underlying mechanisms, and to assess potential benefits in dogs with more severe disease or PLE.

## Off-label antimicrobial declaration

Authors declare no off-label use of antimicrobials.

## Institutional animal care and use committee or other approval declaration

Authors declare no institutional animal care and use committee or other approval was needed.

## Appendix

### Grouping

Group A consisted of patients that had previously undergone non-hydrolyzed or non-single-source novel dietary trials, such as highly digestible or maintenance diets. Group B comprised patients that received dietary trials featuring at least one hydrolyzed commercial or single-source novel protein diet.

### Prior dietary trials

Prior diet trials could involve highly digestible options, such as commercial or homemade gastrointestinal diets, with or without decreased fat content, single-source novel protein, or hydrolyzed protein diets.

### Data collection and time points

Data for each patient were collected at baseline (T0), 1 month (T1), and 3 months (T2) after the start of the study. At baseline (T0), all patients underwent a comprehensive clinical evaluation, including signalment, dietary and medical history, detailed documentation of gastrointestinal clinical signs, duration from the onset of clinical signs, BW, and BCS. Additionally, the CIBDAI score was calculated. Based on the CIBDAI scoring index, the total composite scores were graded as follows: 0-3 as “clinically insignificant disease”, 4-5 as “mild disease,” 6-8 as “moderate disease,” and 9 or more as “severe disease.”^18^ Additionally, the frequency of clinical signs was recorded, along with diarrhea type classification (small, large, or mixed), a complete laboratory evaluation (CBC, serum biochemistry, electrolyte concentrations, and serum cobalamin concentration), and gastrointestinal histopathology, if available.

In addition to the CIBDAI score, at T1 and T2, patients were further categorized into 4 subgroups based on the recurrence pattern of clinical signs: “unchanged,” for patients that experienced clinical signs or flare-ups with the same frequency as before the diet trial; “worsened,” for patients undergoing a higher frequency of clinical signs than at baseline; “improved,” for patients that continued to experience clinical signs or flare-ups, but less frequently, occurring once or less per month; and “resolved” for patients that no longer exhibited any clinical signs or flare-ups at any time point (Table 2). Follow-up assessments (T1 and T2) included BW, BCS, recording of gastrointestinal clinical signs, CIBDAI scores, recurrence patterns of clinical signs, type of diarrhea, and ongoing diet acceptability assessments.

### Secondary outcome measures

Patients were assessed for diet acceptance at each time point based on the caregiver’s perception of their dog’s willingness to accept the diet, and were classified as excellent, moderate, or poor. Dogs classified as excellent exhibited a robust appetite and consistently consumed their entire calculated energy allowance. Those rated as moderate demonstrated a decreased appetite compared with previously fed diets, but still met their caloric requirements. Dogs that failed to consume their calculated intake were categorized as having poor diet acceptability. Patients that completely refused the diet were included in the acceptability assessment but excluded from the clinical efficacy analysis.

### Statistical analysis

Data were extracted into Microsoft Excel (version 16.78) and transferred to R (version 4.4.2) using the R Commander graphical user interface (version 2.9-5) for statistical analysis. Descriptive statistics for categorical variables were presented as frequencies and percentages. Continuous variables were summarized using the median and range, or the mean ± SD, as appropriate, and their distributions were assessed using the Shapiro–Wilk test. Comparisons of categorical variables were performed using the Chi-squared test or Fisher’s exact test, as appropriate. For continuous variables, Student’s *t-*test was used for normally distributed data after verification of homogeneity of variances with Bartlett’s test; otherwise, the Wilcoxon rank-sum test was applied. When variables were compared across response T1 categories, one-way analysis of variance with Tukey’s honesty significant difference post hoc test was used when normality and homogeneity of variances assumptions were met; otherwise, the Kruskal–Wallis test with Dunn’s pairwise comparisons and Holm adjustment was employed. Repeated measures were evaluated at baseline (T0) and at T1. The paired Student’s *t-*test was used for normally distributed data, and the Wilcoxon signed rank test was used for non-normally distributed data. A *P*-value < .05 was considered statistically significant.

### Baseline diagnostic evaluation

At baseline, patients underwent a CBC, serum biochemistry profile, and abdominal ultrasonography, and, when clinically indicated, a baseline serum cortisol concentration or an adrenocorticotropic hormone stimulation test. Additional diagnostic tests, including trypsin-like immunoreactivity, abdominal radiographs, infectious disease testing, and bile acid stimulation test, were performed at the attending clinician’s discretion when clinically indicated.

### Medications allowed previously and during the dietary trial

Exclusion criteria included treatment with glucocorticoids or other immunosuppressive treatments within 2 weeks before starting the diet trial. Antibiotic or probiotic treatments, other supplements such as dietary fiber, were discontinued before the dietary trial began and not introduced during the study evaluation time-points, but cobalamin supplementation was permitted. Concurrent medications unrelated to the gastrointestinal disease (eg, cardiovascular medications) were permitted if they were started before enrollment and there was no clinical suspicion that they contributed to gastrointestinal signs.

## Abbreviations

ARE: antibiotic-responsive enteropathy
BCS: body condition score
BW: body weight
CE: chronic enteropathy
CIBDAI: Canine Inflammatory Bowel Disease Activity Index
CCECAI: Canine Chronic Enteropathy Clinical Activity Index
CR: complete response
cPLI: canine pancreatic lipase
FRE: food-responsive enteropathy
IRE: immunosuppressant-responsive enteropathy
NR: non response
NRE: non-responsive enteropathy
PLE: protein-losing enteropathy
PR: partial response
